# Noninvasive evaluation of pulmonary hypertension using the second heart sound parameters collected by a mobile cardiac acoustic monitoring system

**DOI:** 10.3389/fcvm.2023.1292647

**Published:** 2023-12-15

**Authors:** Jingjuan Huang, Weiwei Zhang, Wenxia Fu, Jiawei Le, Yiding Qi, Xumin Hou, Xin Pan, Ruogu Li, Ben He

**Affiliations:** ^1^ Department of Cardiology, Shanghai Chest Hospital, Shanghai Jiao Tong University School of Medicine, Shanghai, China; ^2^ Department of Cardiac Function, Shanghai Chest Hospital, Shanghai Jiao Tong University School of Medicine, Shanghai, China

**Keywords:** acoustic cardiography, the second heart sound frequency, a mobile cardiac acoustic monitoring system, pulmonary hypertension (PH), estimated pulmonary artery systolic pressure (ePASP)

## Abstract

**Background:**

Pulmonary hypertension (PH) is linked to higher rates of morbidity and mortality worldwide. Early diagnosis of PH is important for clinical treatment. The estimated pulmonary artery systolic pressure (ePASP ≥ 35 mmHg) measured by echocardiography helps screen PH patients. In this paper, we report a novel PH screening method through a mobile cardiac acoustic monitoring system.

**Methods:**

In the retrospective study, patients admitted to our hospital between January 2022 and April 2023 were classified into PH and control groups using ePASP and compared with acoustic cardiographic parameters. According to ePASP, PH severity was classified as mild, moderate, and severe. We analyzed the first and second heart sound (S1, S2) characteristics, including amplitude (S1A, S2A), energy (S1E, S2E), and frequency (S1F, S2F).

**Results:**

The study included 209 subjects, divided into PH (*n* = 121) and control (*n* = 88) groups. Pearson correlation analysis confirmed the positive correlation between S2F and ePASP. The diagnostic performance of S2F as assessed by the area under the ROC curve was 0.775 for PH. The sensitivity and specificity of diagnosing ePASP ≥ 35 mmHg when S2F ≥ 36 Hz were found to be 79.34% and 67.05%, respectively, according to ROC analysis. Severity classification was performed using S2F, the area under the ROC curve was 0.712–0.838 for mild PH, 0.774–0.888 for moderate PH, and 0.826–0.940 for severe PH.

**Conclusions:**

S2F collected by the mobile cardiac acoustic monitoring system offers a convenient method for remote PH screening, potentially improving PH management and outcomes.

## 1. Introduction

Pulmonary hypertension (PH) is a devastating progressive disease characterized by elevated pulmonary artery pressure along with pulmonary vascular resistance that ultimately leads to right heart failure and premature death (1). With the expansion of PH treatment options, the requirement for precise and non-invasive techniques to allow regular and safe estimation of pulmonary arterial pressure (PAP) has increased. PH is defined as an increase in mean pulmonary arterial pressure (mPAP) beyond the threshold of 20 mmHg during a resting state, evaluated using right heart catheterization (RHC) (2). RHC is recognized as the gold standard for the diagnosis of PH; nonetheless, the procedure is complex, invasive, and carries inherent risks, making it unsuitable for early diagnosis, routine screening, and sustained, longitudinal monitoring (3). Transthoracic echocardiography (TTE) is advocated as a non-invasive substitute for the screening of PH. The assessment of estimated pulmonary artery systolic pressure (ePASP) via TTE has demonstrated a robust association with measurements obtained through RHC (4, 5). Resting-state ePASP values of ≥35 mmHg measured by echocardiography indicate PH, with severity ranging from mild (35–45 mmHg) to moderate (45–60 mmHg) to severe (>60 mmHg) (6). Nonetheless, accurate TTE results require experienced and skilled staff. Consequently, it is necessary to develop new non-invasive methods to frequently and accurately assess ePASP and screen potential PH patients.

Acoustic cardiography is a noninvasive, safe, inexpensive, continuous monitoring method that quantifies the state of the heart and lung by synchronously analyzing phonocardiogram (PCG) and electrocardiogram (ECG) (7). In recent years, studies on acoustic cardiography have suggested that the characterization of the second heart sound (S2) can be used to estimate PAP (8, 9). The S2 consists of two audible components, the pulmonic closure sound (P2) and the aortic closure sound (A2). Thus, the S2 component is generated by hemodynamic events that occur immediately following the closure of the aortic and pulmonary valves. The vibrations of S2 occur at the end of ventricular contraction, identifying the beginning of ventricular relaxation and the end of mechanical contraction (10). As PAP increases, a notable elevation emerges in the pulmonary component of S2, establishing an interdependent relationship between PAP and the acoustic characteristics of heart sounds (8). However, the relationship between ePASP and S2 is not yet fully clarified, and whether the cut-off value of S2 can be applied to screen patients with PH remains to be investigated.

The objective of this study is to evaluate the correlations between ePASP and S2 using a mobile cardiac acoustic monitoring system, and to determine the value of S2 features in PH screening.

## 2. Methods

### 2.1. Study participants and design

This was a retrospective observational cohort study. Based on TTE findings, patients admitted to our ward were consecutively selected from January 2022 to April 2023 and divided into two groups as per the ePASP: PH group (ePASP ≥35 mmHg) and control group (ePASP <35 mmHg). This cut-off value was based upon the values suggested by the American Academy of Family Physicians (AAFP), which proposes that an ePASP of 35–40 mmHg or greater on echocardiography is suggestive of PH (6). The demographic and clinical baseline data were recorded for all subjects, including age, gender, medical history, the types of PH, and the severity of PH. Prior to enrollment, all participants voluntarily signed written informed consent. The study protocol was reviewed and approved by the Ethics Committee of the Shanghai Chest Hospital of Shanghai Jiao Tong University and conducted in accordance with the Declaration of Helsinki.

### 2.2. Inclusion and exclusion criteria

Inclusion criteria were patients aged ≥ 18 years hospitalized due to cardiovascular diseases. All subjects underwent echocardiography and acoustic cardiography on the same day. Participants were excluded from this study if they met any of the following criteria: severe valvular heart disease, severe ventricular arrhythmia, acute coronary syndrome, severe liver dysfunction, severe chronic obstructive pulmonary disease, constrictive pericarditis, end-stage renal failure, and psychological issues limiting adherence.

### 2.3. Acoustic cardiography

**Figure 1:**
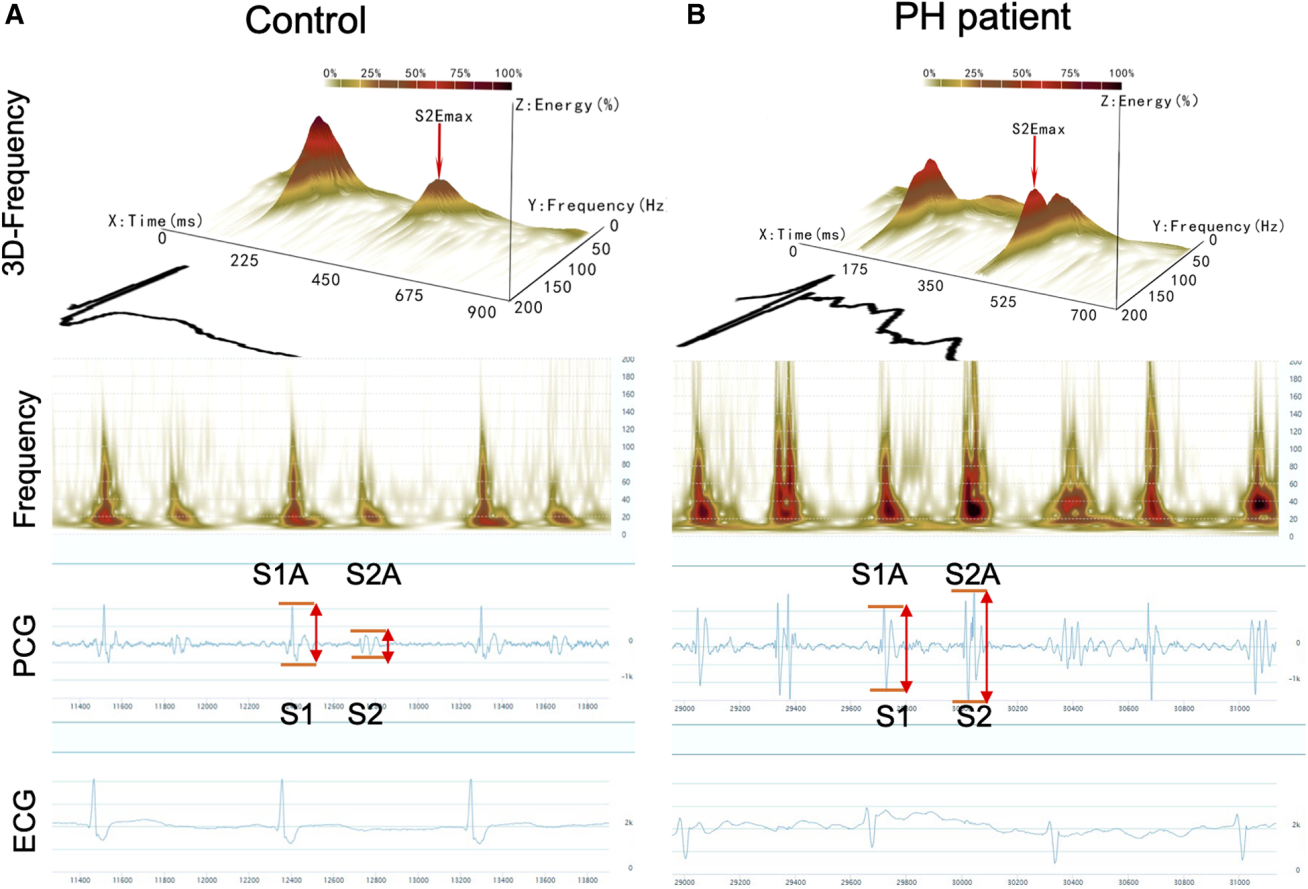
Acoustic cardiographic output for a control patient (**A**) and a patient with PH (**B**) showing frequency, PCG, and ECG. The scalogram time-frequency representation shows frequency on the vertical axis on a logarithmic scale from 0 to 200 Hz and time on the horizontal axis. The squared magnitude of the wavelet transform energy at each frequency is represented by colors ranging from light yellow to deep red. S1, the first heart sound; S2, the second heart sound; S1A, the first sound amplitude; S1E, the first sound energy; S1F, the first sound frequency; S2A, the second sound amplitude; S2E, the second sound energy; S2F, the second sound frequency.

Acoustic cardiography was performed using the WENXIN® device (Wenxin Tech. and Bayland Scientific, Beijing, China and California, USA), and the details have been described previously (11). The device can simultaneously record PCG, ECG and frequency from the precordial V4 locations after patients have rested in a supine position for 5–10 min. The parameters of the first and second heart sounds (S1and S2) were analyzed, including amplitude (S1A, S2A), energy (S1E, S2E), frequency (S1F, S2F), S2/S1 amplitude ratio (S2/S1A), S2/S1 energy ratio (S2/S1E), and S2/S1 frequency ratio (S2/S1F) were measured by an automatic analysis software. The heart sound frequency is the frequency of maximal amplitude, which is obtained by the wavelet transform of the PCG signal (11). The sound spectrogram is a visual representation of an acoustic signal, with varying amplitude over time at different frequencies (10). Figures 1A,B shows the typical acoustic signals and spectral displays from a control and a PH patient. The heart sound audio of two patients is stored in Supplementary Videos S1, S2.

### 2.4. Estimated pulmonary arterial systolic pressure (ePASP)

TTE was used to detect ePASP by measuring the tricuspid regurgitation velocity (TRV) doppler signal (Vivid E95 or Vivid E9, GE Medical Systems, Horten, Norway). Briefly, ePASP was determined by calculating the estimated trans-tricuspid regurgitation gradient (equivalent to 4 × TRV^2^) and adding the estimated right atrial pressure (12). TTEs were reviewed by an experienced echocardiographer blinded to all clinical data and acoustic cardiography findings.

### 2.5. Statistical analysis

The SPSS software package (Version 22.0, SPSS, Chicago, IL, USA) was used to perform statistical analysis. Descriptive statistics include the numbers and percentages for categorical data, and continuous data was represented by the mean ± standard error (SE). We tested the null hypothesis for any difference between groups using the one-way ANOVA or unpaired *t*-test for continuous data and chi-square analysis for dichotomous data. Univariate and multivariate logistic analyses were used to determine the predictive factors of heart sound parameters related to elevated ePASP. Linear logistic regression was performed to test the relationship between ePASP and the heart sounds parameters. Receiver operating characteristic (ROC) curve analysis was performed to explore the predictive value of heart sound parameters in detecting different degrees of ePASP elevation. The areas under the curve (AUC) and the optimal cut-off value of each parameter with the highest sensitivity and specificity were obtained from ROC analysis. Intraclass correlation coefficient (ICC) was calculated between ePASP measured by echocardiography and acoustic sound characteristics. All results were considered statistically significantly different at *P*-value < 0.05.

## 3. Results

### 3.1. Comparison of baseline clinical characteristics, ePASP in PH patients and controls

A total of 209 adult patients were included in this retrospective study between January 2022 and April 2023. Participants were divided into the PH group (*n* = 121) and control group (*n* = 88), according to whether the ePASP ≥ 35 mmHg by transthoracic echocardiography or not. Patients' baseline demographic characteristics and ePASP were presented in Tables 1, 2.

**Table 1:** Baseline clinical characteristics and ePASP of PH patients and controls.

Baseline clinical characteristics	PH (*n* = 121)	Controls (*n* = 88)	*P*-value
Male [*n* (%)]	45 (37.19%)	49 (55.68%)	0.008
Age (years)	67.55 ± 1.39	66.10 ± 1.40	0.474
BMI (kg/m^2^)	21.75 ± 0.25	22.24 ± 0.25	0.188
Hypertension [*n* (%)]	54 (44.6%)	40 (45.5%)	0.906
Diabetes [n (%)]	19 (15.7%)	13 (14.8%)	0.854
Coronary artery disease [*n* (%)]	27 (22.3%)	15 (17.00%)	0.348
Atrial fibrillation	46 (38.0%)	23 (26.10%)	0.071
NYHA class	2.45 ± .056	1.97 ± .069	<0.001
BNP (pmol/L)	417.77 ± 27.17	226.70 ± 19.64	<0.001
ePASP (mmHg)	50.26 ± 1.51	30.02 ± 0.32	<0.001

Values are given as mean ± SE unless otherwise indicated.

PH, pulmonary hypertension; LVEF, left ventricular ejection fraction; ePASP, estimate pulmonary artery systolic pressure; BMI, body mass index; BNP, B-type natriuretic peptide.

**Table 2:** Baseline clinical characteristics of PH patients.

Baseline clinical characteristics	PH (*n* = 121)
Clinical classification of PH	
Group 1	17
Group 2	96
Group 3	1
Group 4	4
Group 5	3
Severity of PH	
Mild (35-45 mm Hg)	63
Moderate (45–60 mm Hg)	34
Severe (>60 mm Hg)	24
Medications	
Endothelin receptor antagonists	16
Calcium channel antagonists	11
Phosphodiesterase 5 inhibitors	4
Prostacyclin analogues	5
Prostacyclin receptor agonist	2
Soluble guanylate cyclase stimulator	1

NYHA, New York Heart Association; PH, pulmonary hypertension; Group 1, pulmonary arterial hypertension (PAH); Group 2, PH due to left-sided heart disease; Group 3, PH due to chronic lung disease; Group 4, chronic thromboembolic PH (CTEPH); Group 5, PH with an unclear and/or multifactorial mechanisms.

### 3.2. Acoustic cardiography measurements

The acoustic characteristics of S1 and S2 in the PH and control groups were shown in Figure 1 and Table 3. Heart rate and PR intervals were similar between the two groups. Patients with PH exhibited a slightly greater QRS duration. Among the PH patients, both the amplitude and frequency of S2 were increased compared with the controls. Hence, the S2/S1 amplitude and frequency ratios were significantly different between the two groups. Although there was no significant difference in S2 energy between the two groups, the S2/S1 energy ratio risen markedly in the PH patients compared with the controls.

**Table 3:** Acoustic cardiographic parameters of PH patients and controls.

Parameters	PH (*n* = 121)	Controls (*n* = 88)	*P*-value
Heart rate (bpm)	70.84 ± 1.16	72.82 ± 1.11	0.234
PR interval (ms)	151.84 ± 2.04	146.38 ± 1.96	0.063
QRS duration (ms)	118.53 ± 1.17	110.74 ± 1.16	<0.001
S1A (mv)	1,222.93 ± 94.59	1,094.78 ± 78.74	0.324
S1E	1,557.22 ± 116.78	1,579.13 ± 131.01	0.901
S1F (Hz)	29.67 ± 1.12	27.45 ± 1.23	0.188
S2A (mv)	979.78 ± 78.12	767.17 ± 69.93	0.044
S2E	1,061.31 ± 80.51	953.18 ± 94.80	0.385
S2F (Hz)	51.42 ± 2.09	32.57 ± 1.27	<0.001
S2/S1A	0.95 ± 0.06	0.78 ± 0.05	0.043
S2/S1E	0.81 ± 0.05	0.65 ± 0.04	0.012
S2/S1F	1.93 ± 0.11	1.34 ± 0.07	<0.001

Values are given as mean ± SE unless otherwise indicated.

PH, pulmonary hypertension; LVEF, left ventricular ejection fraction; ePASP, estimate pulmonary artery systolic pressure; S1A, the first sound amplitude; S1E, the first sound energy; S1F, the first sound frequency; S2A, the second sound amplitude; S2E, the second sound energy; S2F, the second sound frequency; S2/S1A, S2/S1 amplitude ratio; S2/S1E, S2/S1 energy ratio; S2/S1F, S2/S1 frequency ratio.

### 3.3. Relationship between acoustic cardiographic parameters and ePASP

**Figure 2:**
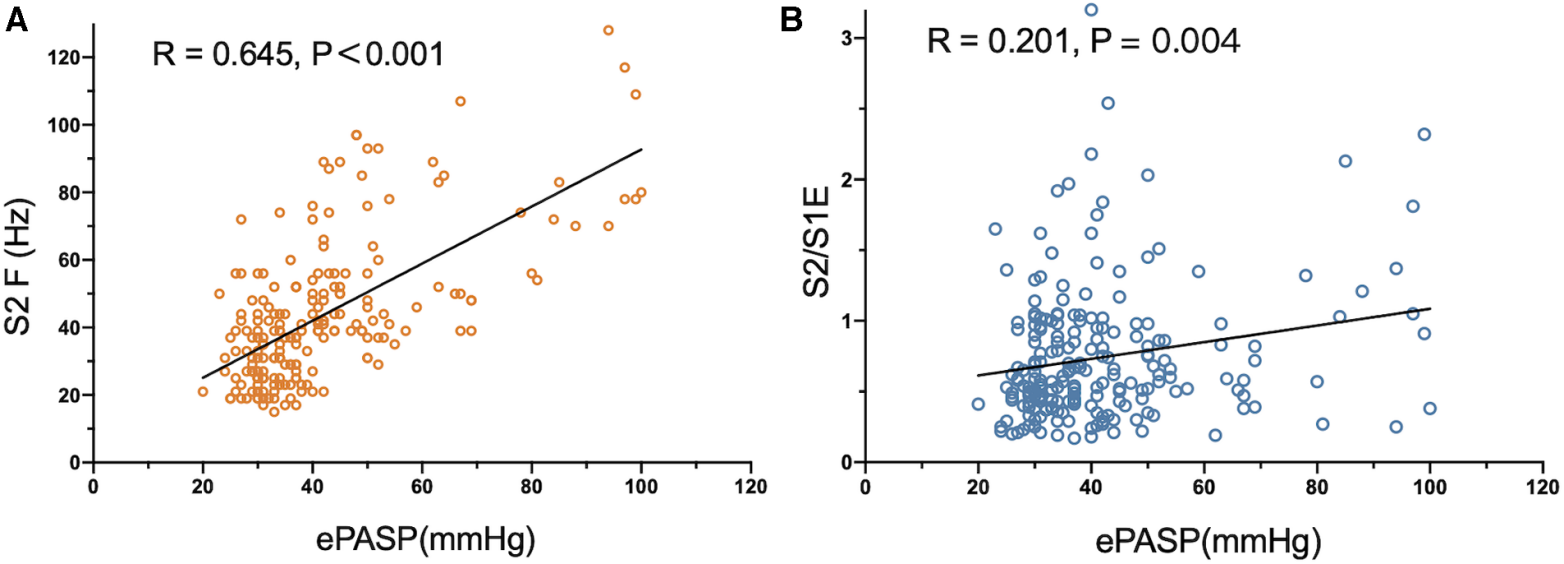
Correlation between S2 frequency, S2/S1 energy ratio and ePASP. ePASP, estimate pulmonary artery systolic pressure; S2F, the second sound frequency; S2/S1E, S2/S1 energy ratio.

As the acoustic cardiographic parameters of S2A, S2F, S2/S1A, S2/S1E, and S2/S1F were remarkably increased in the PH group, we investigated the relationship between ePASP and acoustic cardiographic variables including S2A, S2F, S2/S1A, S2/S1E, and S2/S1F across the cohort (Table 4). S2F and S2/S1E were independent multivariate predictor for ePASP, and S2F performed better than S2/S1E. As shown in Figure 2A, S2F was significantly correlated with increasing the severity of ePASP (*R* = 0.645, *P* < 0.001). In contrast to S2F, we observed that the correlation between S2/S1E and ePASP was only 0.201, *P* = 0.004 (Figure 2B).

**Table 4:** Multiple logistic regression analysis for elevated ePASP predictors of acoustic cardiographic parameters.

Parameters	Odds ratio	95% confidence limits	*P*-value
S2A, every 1mv increase	1.000	1.000–1.001	0.466
S2F, every 1 Hz increase	1.076	1.043–1.111	<0.001
S2/S1A, every 1unit increase	0.320	0.790–1.298	0.111
S2/S1E, every 1unit increase	5.780	1.092–30.593	0.039
S2/S1F, every 1unit increase	1.068	0.583–1.956	0.832

PH, pulmonary hypertension; ePASP,estimate pulmonary artery systolic pressure; S2A, the second sound amplitude; S2F, the second sound frequency; S2/S1A, S2/S1 amplitude ratio; S2/S1E, S2/S1 energy ratio; S2/S1F, S2/S1 frequency ratio.

### 3.4. S2 frequency has strong diagnostic accuracy in identifying different degrees of PH

**Figure 3:**
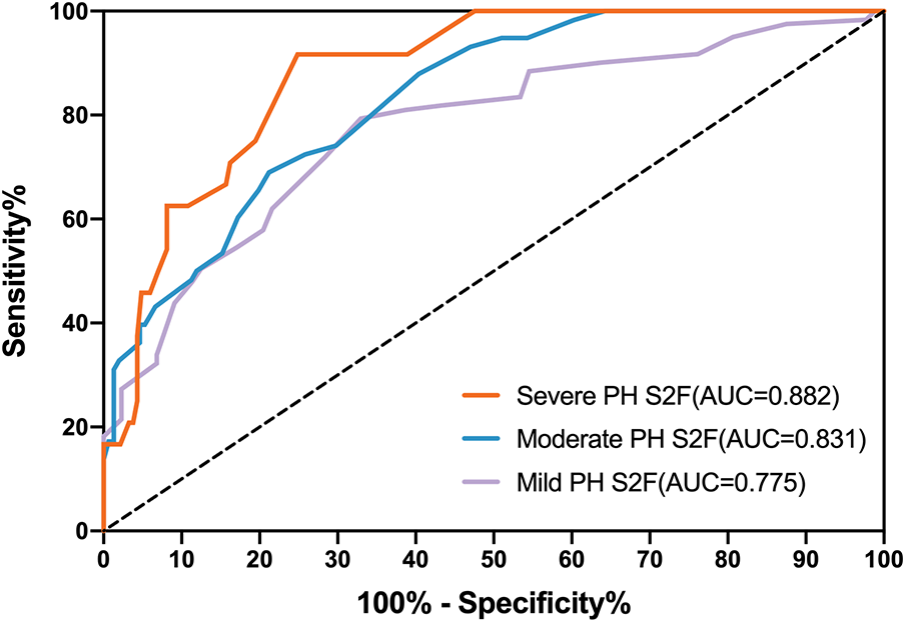
Receiver operator characteristic curves (ROC) for S2F as predictors of different severity of PH. PH, pulmonary hypertension; ePASP, estimate pulmonary artery systolic pressure; S2F, the second sound frequency.

We assessed the predictive performance of S2 frequency as a cut-off value to detect PH (ePASP ≥ 35 mmHg) by constructing ROC curves using echocardiographic data from 121 PH cases and 88 controls. Figure 3 showed that the AUC ROC curve for S2F was 0.775 (*P* < 0.001), indicating the ability of S2F to identify PH (ePASP ≥ 35 mmHg). The optimum cut-off S2F for distinguishing PH was 36 Hz (sensitivity 79.34%, specificity 67.05%, likelihood ratio 2.408) according to Table 5. The ICC was 0.62 (95% confidence interval, 0.53–0.70) between echocardiography estimated ePASP and acoustic cardiography measured S2F.

**Table 5:** Diagnostic accuracy of S2F by the severity of PH.

Severity of PH	ePASP (mmHg)	AUC	95% Confidence interval	Cut-off value	Sensitivity	Specificity	PPV	NPV
Mild	39.30 ± 0.35	0.775	0.712–0.838	36	0.793	0.671	0.768	0.702
Moderate	50.12 ± 0.59	0.831	0.774–0.888	45	0.690	0.788	0.556	0.869
Severe	79.25 ± 2.83	0.883	0.826–0.940	47	0.917	0.751	0.324	0.986

Values are given as mean ± SE unless otherwise indicated. PH, pulmonary hypertension; ePASP, estimate pulmonary artery systolic pressure; AUC, area under the curve S2F, the second sound frequency; PPV, positive predictive value, NPV, negative predictive value.

Diagnostic accuracy using S2F for the three degrees of PH through ROC analysis was presented in Table 5. S2F correctly identified mild PH from the other levels with the AUC range from 0.712 to 0.838, and sensitivity and specificity ranges from 0.713 to 0.856 and from 0.567 to 0.760, respectively. For moderate PH identification by S2F, the AUC range was from 0.774 to 0.888, with sensitivity and specificity varying from 0.562 to 0.794 and from 0.716 to 0.846, respectively. The AUC range for identifying severe PH through S2F was 0.826–0.940, with sensitivity ranging from 0.742 to 0.985 and specificity ranging from 0.684 to 0.881.

## 4. Discussion

As far as we know, this study first presents the evidence that analyzing S2 frequency collected by the mobile cardiac acoustic monitoring system is a reliable and speedy diagnostic tool for screening PH. Our retrospective cohort study explored the quantitative correlation between S2 acoustic features and PH severity. The frequency of S2 was observed to rise in the PH group, concomitant with increased ePASP levels. Moreover, the diagnostic cut-off value for S2 frequency increases with degree of ePASP elevation among the PH patients.

### 4.1. Increased loudness of S2 is a typical feature of heart sounds in patients with PH

The prevailing and widely accepted theory regarding the origin of heart sounds is the “cardiohemic model”. According to this paradigm, the sounds are produced by the vibration of the entire structure and internal components of the heart (13). This vibration is stimulated by specific cardiac valve closure, with the mitral valve governing the genesis of S1 and the combined interaction of the aortic and pulmonic valves dictating the origin of S2. S2 arises from a complex interplay involving the dynamic processes accompanying the relaxation of the left and right ventricles, the closure of the aortic and pulmonary valves, and the compliance of the aorta and main pulmonary artery (14, 15). The onset of S2 implies the end of mechanical ventricular systole as well as the beginning of ventricular diastole. The aggravation of the pulmonic component of S2 reflects strong closure of the pulmonary valve, suggesting RV pressure overload. Considering A2 and P2 of S2 are usually very close in the time axis, clinical indicators of PH can be concluded as the increased loudness of P2 of S2 and increased transmission of P2 to the cardiac apex. However, it should be noted that a small percentage of cases where only increased A2 loudness causes false positives (i.e., LV systolic dysfunction or left-to-right shunting). The presence of abnormal auscultatory observations manifested as notable frequency was documented in the Primary Pulmonary Hypertension Registry supported by the National Institutes of Health. Specifically, a discernible augmentation in the second component (P2) of S2 was documented in a substantial majority, accounting for 93%, among a cohort of 187 individuals diagnosed with PH (16). However, conventional cardiac auscultation is a challenging process and can be impacted by various factors, such as patient characteristics and the clinical experience of the physicians, leading to inconsistent results obtained by different observers (17, 18). In this research, the wavelet-based signal processing analysis was utilized for handling continuous digital acoustic data, which yielded the measurements of amplitude, energy, and frequency, thereby resulting in more objectively and realistically reflecting characteristics of the heart sounds.

### 4.2. S2 frequency, as an inherent characteristic of sound waves, can better reflect the changes in PH

Using acoustic cardiography, the auscultatory heart sounds were collected, quantified, and recorded as sound waveforms. The waveform characteristics of increased loudness of S2 were then reflected as amplitude, frequency, and energy (10, 19). The sounds' amplitude depends on the force generated by valve closure, which in turn relies on the pressure gradient across the valve during the closing. The volume and contents of the heart are also important, in determining the resonance of the cardiohemic system (20). As previously demonstrated, the frequencies of heart sounds can be determined by the volume of the vibrating mass and the tension produced in the walls of the heart and great vessels (21). As the pressure rises, the resonant frequency also increases. The energy value calculated based on the heart sound waveform is coupled with the influence of both amplitude and frequency factors. The greater the amplitude, the higher the energy value; and the greater the frequency, the higher the energy level (22). The amplitude varies with the body weight, acquisition position, and posture. However, as an inherent feature of sound waves, the frequency does not change with the influence of the acquisition conditions or the environment, thus can better reflect the changes in heart sounds.

The subjects were classified into the PH and control groups, depending on whether ePASP was greater than 35 mmHg, which was consistent with the current definition and diagnostic recommendations for PH (4). There was a significant statistical difference in the echocardiography-measured ePASP between the two groups (50.26 ± 1.51 vs. 30.02 ± 0.32 mm Hg; *P* < 0.001) in Table 1. In the current study, we analysed the acoustic characteristics of S1 and S2 in both groups, and the results indicated that S1A, S1E, S1F, and S2E were similar between the PH and control groups (see Table 3).

The high-frequency components of S2 may be caused by increased wall tension in the right heart and pulmonary artery. In Table 3, we found a significant increase in the frequency of S2 in patients with PH (51.42 ± 2.09 vs. 32.57 ± 1.27 Hz, *P* < 0.001), which aligned with the previous observation of S2 frequently being perceived as abnormal in this condition (23). It is encouraging to distinguish subjects with PH from patients with normal ePASP by extracting the constituent features of S2 in the frequency domain.

It has been shown that the amplitude of heart sounds is directly related to myocardial contractility in adult patients undergoing stress testing (24). In our study, the S2 amplitude increased in PH patients (979.78 ± 78.12 vs. 767.17 ± 69.93mv; *P* = 0.044). There was a gradual increase in the size of the amplitude waveforms associated with PH as shown in Figure 1B. This phenomenon can be effortlessly clarified by the established impact of elevated ePASP on myocardial contractility. The increased rate of PAP during right ventricle during systole may lead to an increased force of tricuspid valve closure, thus resulting in a higher amplitude of S2.

The control group had a relatively small sample size, and there was an absence of age or sex matching with the PH group. Nevertheless, we adjusted for possible intersubject anthropomorphic differences by calculating the S2/S1 amplitude, frequency, and energy ratios. The results of this study demonstrated that the S2/S1A, S2/S1F, and S2/S1E in the PH group were markedly higher than that in controls, and all the differences were statistically significant (Table 3).

### 4.3. S2 frequency is reliable and effective in assisting PH screening

We examined the correlation between ePASP and digitally recorded precordial acoustic characteristics in PH and control groups. In this study, we found S2A, S2/S1A, and S2/S1F were not strong indicators of PH. As shown in Table 4, only S2F and S2/S1E were considered significant independent predictors of PH based on the multivariate analysis. Furthermore, the Pearson correlation analysis indicated that ePASP correlated more strongly with S2F than with S2/S1E in Figure 2. S2F and ePASP had a significantly positive correlation (*R* = 0.645, *P *< 0.001). This can be explained based on the differences in mechanism. The amplitude is mainly determined by the closing force of the heart valves, while the frequency can be affected by the closing force, cardiac volume, and the resonance frequencies of the heart and the great vessels. Therefore, differences in pressure and intravascular volume could lead to more variation of frequency than amplitude and energy in PH patients, thus resulting in a more significant statistical correlation between S2F and ePASP. Other scholars have discussed the use of frequency components as measures of intravascular pressure and wall tension (25, 26). However, to our knowledge, there have been no reports that frequency can characterize the PAP state of the heart. In addition, we found a reciprocal link between S2 and S1 that may relate to ventricular interaction in PH patients. We observed that S2/S1E was an independent multivariate predictor of ePASP, which was in line with other researches examining S2 acoustic properties and pulmonary artery pressure determined from TTE or RHC (27, 28). The QRS duration was shorter in subjects with PH (118.53 ± 1.17 vs. 110.74 ± 1.16 ms; *P* < 0.001). A widened QRS duration or delayed right heart conduction time might influence the splitting interval between A2 and P2 but not the frequency of S2. Age, BMI, and concurrent disorders were not statistically significantly different between the two groups, indicating that these factors were not the cause of the disparities in S2 frequency.

These findings support the established conception that elevated PAP leads to forceful closure of the pulmonic valve and indicate the reliability and validity of S2F in assisting PH screening.

### 4.4. Different cut-off values of S2 frequency in patients with different severity of PH

We found significant variations in S2F regardless of the severity of PH. According to Figure 3 and Table 5, the accuracy for S2F's PH severity varied from 0.712 to 0.838 for mild PH, 0.774–0.888 for moderate PH, and 0.826–0.940 for severe PH. These findings were comparable to those obtained using echocardiographic monitoring, where it was noted that the diagnosis accuracy for PH type ranged from 0.689 to 0.8 (29–31).

Due to the relatively high NPV values found in severe PH (NPV = 0.9858), S2F may also have the potential to identify individuals with less severe PH (ePASP<60 mmHg). There are various situations when S2F can be employed in clinical settings. For instance, an individual who has S2F greater than or equal to 47 Hz is suspected to have severe PH. S2F greater than or equal to 37 Hz, and smaller than 45 Hz indicates possible mild PH (Table 5).Outpatient use of S2F as a screening tool can selectively separate patients for further examinations like echocardiographic measurement or RHC.

### 4.5. The superiority of acoustic cardiography in evaluating PH

The advantages of applying acoustic cardiography in screening for PH patients include relatively low cost, non-invasiveness, ease of use, and early identification of elevated ePASP.

Since acoustic cardiographic parameters can be easily obtained through non-invasive devices equipped with ECG and PCG sensors (32), they can serve as useful indicators for screening PH. Patients can conveniently record their daily acoustic cardiography on their own terms due to the portability and usability features of these devices. The long-term trend of these data provides valuable insight to detect PH deterioration prior to adverse events, allowing physicians to consider preventative interventions proactively. However, further evaluation of this technology is required to assess its actual efficacy in remote monitoring of diverse patient ranges.

### 4.6. Limitations of the study

There are still some limitations to acoustic cardiography. First, the quality of data may be compromised by both endogenous and exogenous noises, such as respiratory and background noise. During the heart sound recording process, keeping quiet and breathing calmly as much as possible helps to ensure high-quality data collection. Second, we only studied the association of ePASP estimated by echocardiography and acoustic variables and did not further investigate the relationship between RHC parameters and heart sound parameters. Finally, the 2022 ESC/ERS Guidelines for the diagnosis and treatment of PH suggested using the peak tricuspid regurgitation velocity (TRV) as a crucial factor to determine the echocardiographic likelihood of PH. A peak TRV above 2.8 m/s could suggest the possible presence of PH; nevertheless, the presence of PH cannot be reliably determined by TRV alone, and ePASP is the most used parameter for screening PH in clinical practice.

## 5. Conclusions

In summary, we demonstrated that S2F was associated with elevated ePASP and had different cut-off points in patients with different severity of PH. Using the mobile cardiac acoustic monitoring system, S2F measurements offer a convenient ambulatory method for PH screening, making it more user-friendly than the standard examination.

## Data Availability

The raw data supporting the conclusions of this article will be made available by the authors, without undue reservation.

## Supplementary material

SUPPLEMENTARY AUDIO S1

The heart sound audio of the control patient.

SUPPLEMENTARY AUDIO S2

The heart sound audio of the PH patient
